# PRomotion Of Physical activity through structured Education with differing Levels of ongoing Support for people at high risk of type 2 diabetes (PROPELS): study protocol for a randomized controlled trial

**DOI:** 10.1186/s13063-015-0813-z

**Published:** 2015-07-02

**Authors:** Tom Yates, Simon Griffin, Danielle H Bodicoat, Gwen Brierly, Helen Dallosso, Melanie J Davies, Helen Eborall, Charlotte Edwardson, Mike Gillett, Laura Gray, Wendy Hardeman, Sian Hill, Katie Morton, Stephen Sutton, Jacqui Troughton, Kamlesh Khunti

**Affiliations:** NIHR Leicester-Loughborough Diet, Lifestyle, and Physical Activity Biomedical Research Unit, Leicester, UK; Diabetes Research Centre, College of Medicine, Biological Sciences and Psychology, University of Leicester, Leicester, LE5 4PW UK; Department of Public Health and Primary Care, University of Cambridge, Institute of Public Health, Forvie Site, Box 113 Cambridge Biomedical Campus, Cambridge, CB2 0SR UK; MRC Epidemiology Unit, University of Cambridge School of Clinical Medicine, Box 285 Institute of Metabolic Science, Cambridge Biomedical Campus, Cambridge, CB2 0QQ UK; Leicester Diabetes Centre, University Hospitals of Leicester, Leicester General Hospital, Leicester, LE5 4PW UK; Social Science Applied to Healthcare Improvement Research (SAPPHIRE) Group, Department of Health Sciences, University of Leicester, 22-28 Princess Road West, Leicester, LE1 6TP UK; School of Health & Related Research (ScHARR), University of Sheffield, Regent Court, 30 Regent Street, Sheffield, S1 4DA UK; Department of Health Sciences, College of Medicine, Biological Sciences and Psychology, University of Leicester, 22-28 Princess Road West, Leicester, LE1 6TP UK; Behavioural Science Group, Primary Care Unit, Institute of Public Health, Forvie Site, University of Cambridge, School of Clinical Medicine Box 113, Cambridge Biomedical Campus, Cambridge, CB2 0SR UK; UK CRC Centre for Diet and Activity Research (CEDAR), MRC Epidemiology Unit, University of Cambridge School of Clinical Medicine, Box 285 Institute of Metabolic Science, Cambridge Biomedical Campus, Cambridge, CB2 0QQ UK

**Keywords:** Prevention, Impaired glucose regulation, Type 2 diabetes, Prediabetes, Walking, Physical activity, Pedometer, Structured education, Primary care, Randomised controlled trial, mHealth

## Abstract

**Background:**

The prevention of type 2 diabetes is recognised as a health care priority. Lifestyle change has proven effective at reducing the risk of type 2 diabetes, but limitations in the current evidence have been identified in: the promotion of physical activity; availability of interventions that are suitable for commissioning and implementation; availability of evidence-based interventions using new technologies; and physical activity promotion among ethnic minorities. We aim to investigate whether a structured education programme with differing levels of ongoing support, including text-messaging, can increase physical activity over a 4 year period in a multi-ethnic population at high risk of diabetes.

**Methods/Design:**

A multi-centre randomised controlled trial, with follow-up at 12 and 48 months. The primary outcome is change in ambulatory activity at 48 months. Secondary outcomes include changes to markers of metabolic, cardiovascular, anthropometric and psychological health along with cost-effectiveness. Participants aged 40–74 years for White European, or 25–74 years for South Asians, with an HbA1c value of between 6.0 and < 6.4 % (42 and 47 mmol/mol) or with a previously recorded plasma glucose level or HbA1c value within the high risk (prediabetes) range within the last five years, are invited to take part in the trial. Participants are identified through primary care, using an automated diabetes risk score within their practice database, or from a database of previous research participants.

Participants are randomly assigned to either: 1) the control group who receive a detailed advice leaflet; 2) the Walking Away group, who receive the same leaflet and attend a 3 hour structured education programme with annual maintenance sessions delivered in groups; or 3) the Walking Away Plus group, who receive the leaflet, attend the structured education programme with annual maintenance sessions, plus receive follow-on support through highly-tailored text-messaging and telephone calls to help to aid pedometer use and behaviour change.

**Discussion:**

This study will provide new evidence for the long-term effectiveness of a structured education programme focused on physical activity, conducted within routine care in a multi-ethnic population in the UK. It will also investigate the impact of different levels of ongoing support and the cost-effectiveness of each intervention.

**Trial registration:**

ISRCTN83465245 Trial registration date: 14/06/2012

## Background

According to the World Health Organisation (WHO), type 2 diabetes mellitus (T2DM) is the third leading cause of mortality globally and represents one of the greatest health challenges for modern society, both in the UK and worldwide [1]. The International Diabetes Federation estimate there are 387 million people living with diabetes, and this is predicted to rise to 592 million by 2035 [2]. The healthcare expenditures associated with T2DM, the commonest form of diabetes, are substantial and having T2DM drastically increases lifetime healthcare expenditures [3].Diabetes currently accounts for approximately 10 % of the total health resource expenditure in the UK and is projected to account for around 17 % in 2035⁄2036, due to a sharply increasing prevalence [4]. Consequently health care organisations have recognised the need for policy and recommendations aimed at prevention at the international, national and regional level. In England this has taken the form of the National Health Service (NHS) Health Checks Programme [5], designed to screen and treat those at risk of vascular disease in adults between 40 and 75 years. The National Institute of Health and Care Excellence (NICE) have also recently issued guidance aimed at the prevention of T2DM in at-risk adults [6].

The status of being at high risk of T2DM refers to an intermediary glucose control category that is outside the normal range but below that needed to diagnose T2DM. This intermediary range has historically been termed ‘prediabetes’ or ‘intermediate hyperglycemia’ and classified through oral glucose tolerance test defined impaired fasting glucose or impaired glucose tolerance [7] . Along with traditional definitions, an International Expert Committee convened by the International Diabetes Federation and NICE now also recognise that HbA1c can be used to identify those at high risk of T2DM in the range of 6.0 % - 6.4 % [6, 8]. Twenty five to 40 % of individuals in these risk categories go on to develop T2DM over a 10 year period and as many as 70 % will eventually develop T2DM over the course of their lifetime [9]. While we recognise there is controversy and lack of consensus in terminology for these categories of glucose dysregulation, we refer to prediabetes henceforth to aid readability.

Lifestyle interventions aimed at weight regulation, dietary modification and increased physical activity have successfully reduced the risk of T2DM in those with prediabetes in diverse settings [10] and are recommended as the first level of intervention for the prevention of T2DM [6, 11]. However, several limitations in the evidence have not been adequately addressed. These include effective interventions to promote physical activity, availability of interventions that are suitable for commissioning and implementation, use of new technologies to increase the scalability of interventions, and the promotion of health behaviour within ethnic minorities. Each of these limitations is discussed below followed by an introduction to how structured education and new technologies may be employed as a solution in the context of the NHS in the UK.

### Physical activity

Although physical inactivity is one of the most important lifestyle determinants contributing to the risk of both T2DM and cardiovascular disease [12–14], there is little direct evidence that previous T2DM prevention programmes have resulted in clinically significant increases in physical activity [15], even when this health behaviour is the primary intervention target [16]. The effectiveness of diabetes prevention programmes could therefore be enhanced if effective, evidence-based strategies for increasing physical activity are developed and evaluated within the context of diabetes prevention in primary care.

### Translation into routine care

Trials of the efficacy of lifestyle interventions in the prevention of T2DM have evaluated highly resource intensive programmes, beyond the level that could be rolled out in the real world given resource limitations and competing clinical needs [17]. Whilst there is mounting evidence internationally that the approach used in efficacy trials can be translated into approaches suitable for implementation in a community or primary care setting [18, 19], there is limited evidence for the long-term effectiveness of these programmes, and evidence-based behaviour change techniques to support the maintenance of behaviour change. In addition, there has been a lack of systematic approaches to translating evidence into practice within the UK setting. Diabetes prevention programmes that are tailored to the resources and infrastructure within primary care and compliant with NICE guidelines are being developed [20], but there is a need to ensure these are thoroughly evaluated over the longer-term.

### Use of new technologies to increase scalability

New technologies such as the internet (eHealth) and mobile phones (mHealth) offer great potential for the delivery of behaviour change interventions, given their scalability, reach, and capacity to offer highly-tailored, interactive behaviour change support. Text messaging interventions have shown promise in supporting medication adherence, physical activity, weight loss, smoking cessation and prevention/management of chronic disease, either as a standalone intervention or in combination with other modes of delivery, e.g. face to face [9, 21–25]. However, these interventions are rarely based on evidence and offer general rather than highly tailored support. There is uncertainty about the long-term effectiveness of theory driven and evidence-based behaviour change interventions using mHealth technology that can be integrated into routine diabetes prevention pathways within primary care [26].

### Ethnicity

In industrialised societies, some minority ethnic groups are known to have a substantially elevated risk of T2DM. South Asians (SAs), the largest ethnic minority in the UK, have prevalence and progression rates for diabetes that are up to four times greater than the general population [27]. This elevated risk of chronic disease is compounded by lifestyle factors, of which the most notable is physical inactivity. SAs residing in the UK have been shown to be substantially less active with lower levels of cardiovascular fitness than the general population [28–30]. This differential in physical activity behaviour and levels of cardiorespiratory fitness has been linked to the increased prevalence of chronic disease and higher rates of insulin resistance seen in SA populations [30, 31]. Therefore SAs represent a priority group in the prevention of T2DM. However, there is limited evidence that diabetes prevention programmes within a European context have been effective at targeting lifestyle behaviours and improving health in minority ethnic groups.

### Structured education for the prevention of T2DM

There is encouraging pilot evidence that the limitations highlighted may be addressed by combining structured education with pedometer use [32]. Structured education refers to educational interventions, generally delivered to small groups, aimed at the promotion of self-management and health behaviour and underpinned by established health behaviour theories, a written curriculum and standardised educator training and quality assurance pathways. Structured education has been widely used as a central part of diabetes management pathways within routine care and has been recommended by NICE since 2003 [33]. The Diabetes Education and Self-Management for Ongoing and Newly Diagnosed (DESMOND) programme is one of the most prominent structured education programmes for people diagnosed with T2DM available nationally to commissioning organisations in the UK and the only programme to undergo a multi-centred randomised controlled trial to quantify effectiveness and cost-effectiveness [34, 35]. The DESMOND trial reported reductions in cardiovascular disease risk profile, reduced depression, enhanced smoking cessation, and weight loss whilst being highly cost-effective at £2092 per QALY gained [34, 35]. Given the existing wide spread infrastructure of the DESMOND model, it has started to be adapted to the prevention arena as a feasible and scalable model for implementing diabetes prevention programmes within primary care and public health [20].

Pilot work concluding with a single-centre randomised controlled trial demonstrated that the approach used to promote self-management in the DESMOND programme can be tailored to those identified with prediabetes in the promotion of physical activity. The Prediabetes Risk Education and Physical Activity Recommendation and Encouragement (PREPARE) structured education programme was found to increase physical activity levels and substantially reduce fasting and post-challenge glucose levels in a multi-ethnic population over 12 months when combined with personalised pedometer use [32]. Given the relevance to healthcare providers and commissioners, it is important to investigate the effectiveness of this approach further when translated into routine clinical care.

Whilst structured education has been identified as a potentially effective method of promoting behaviour change in the prevention of T2DM, guidelines recommend the provision of regular ongoing support [6, 36]. It is essential to develop sustainable and scalable approaches, such as mHealth, to enhance and reinforce the aims of structured education programmes or other face-to-face behaviour change interventions.

Given the above considerations, there is an identified need to develop and evaluate the use of structured education as a tool for promoting health behaviour change in those with prediabetes with tailored, ongoing support through low-resource approaches. Here we test whether the approach used in the PREPARE structured education programme, which has proven efficacy [32, 37], can promote long-term changes to physical activity and whether the programme’s effectiveness is enhanced through the use of a follow-on support programme combining pedometer self-monitoring with tailored text-messaging and telephone calls.

### Aims and objectives

1. To investigate whether an intervention to support physical activity change and maintenance, offered to an ethnically diverse population with prediabetes, can lead to sustained increases in physical activity over four years.
2. To investigate the effectiveness of the intervention when delivered at two levels of intensity, with and without follow-on support that enhances self-monitoring with pedometers through tailored text-messaging and telephone calls.
3. To investigate the effect of the intervention within White Europeans and South Asians sub-groups.
4. To conduct a within-trial and long-term economic evaluation of both intervention conditions using the costs and benefits arising from the study, rates of progression to T2DM, biomedical outcomes, NHS resource use, and quality-of-life.

## Methods/Design

### Study design

The study comprises two phases: a development and piloting phase of the text-messaging support for pedometer use and a multi-centre randomised controlled trial. The development phase has been submitted for publication elsewhere, while the present article describes the trial phase.

**Fig. 1:**
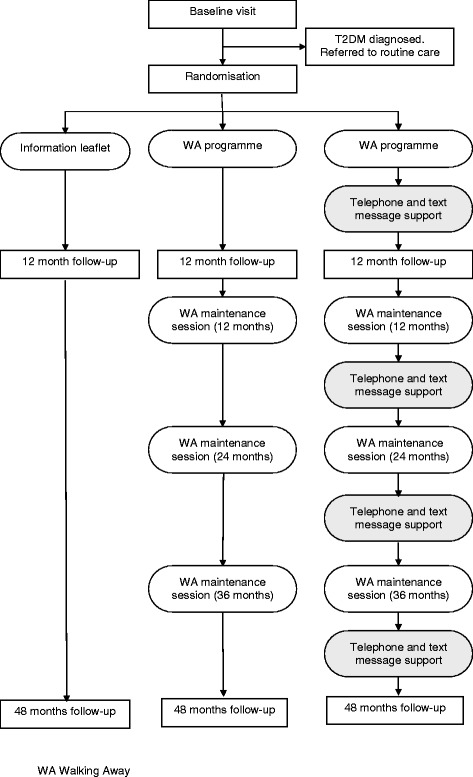
Flow of participants through the study

The trial is a multi-centre randomised controlled trial comparing two modes of physical activity intervention with a control condition. The study is designed to adhere to internationally recognised criteria for developing complex interventions [38]. The design of the study and flow of participants is described in Fig. 1. The study is being run across two centres, Leicester and Cambridge, UK. A total of 1308 participants will be recruited, with 66 % (n = 863) recruited from Leicester. The aim is to have at least 25 % (n = 327) of the total cohort from a South Asian ethnic origin to allow for increased generalisability and the ability to stratify results by ethnicity (see Sample Size).

The trial is sponsored by the University of Leicester and ethical approval was granted by the NHS National Research Ethics Service, East Midlands Committee, which co-ordinates ethical permissions across the following study and recruitment sites:

- University Hospitals of Leicester NHS Trust
- Leicester City Clinical Commissioning Groups (CCG)
- West Leicestershire CCG
- East Leicestershire and Rutland CCG
- University of Cambridge
- MRC Epidemiology Unit
- Cambridge and Peterborough CCG
- Cambridge University Hospitals NHS Foundation Trust

### Recruitment of participants

The primary method of recruitment is through the NHS Health Checks programme. The NHS Health Check is a screening programme run in the UK, designed to identify and treat vascular disease risk (heart disease, stroke, diabetes, and kidney disease) in all individuals aged 40–74 years [5]. As part of this, many primary care practices now have HbA1c or fasting glucose values recorded for their patients. The research teams in both Leicester and Cambridge are working in collaboration with practices that are providing the Health Checks programme to recruit those found with prediabetes, and who are not currently receiving a systematic diabetes prevention pathway. To help with this process, recruited practices are trained to run an established automated diabetes risk score within their practice database [39]. A function within the risk score uses a Morbidity Query Information Export Syntax (MIQUEST) search to identify all individuals who have had a previous blood glucose or HbA1c result recorded in the prediabetes range (see Table 1) over the previous five years [39]. In Cambridge, participants meeting the inclusion criteria are also being recruited from existing population level research databases.

**Table 1:** Categories of glycaemic control used for this trial

	Normal Glycaemia	Prediabetes**		Type 2 Diabetes
	Upper value	Lower value	Upper value	Lower value
HbA1c (%)*	<6.0	≥6.0	<6.5	≥6.5
HbA1c (mmol/mol)*	<42	≥42	<48	≥48
Fasting plasma glucose (mmol/l)*	<5.5*	≥5.5	<7.0	≥7.0
2-hour post challenge glucose (mmol/l)	<7.8	≥7.8	<11.1	≥11.1

*NICE guidelines (2012) [6]

**Levels within this range within the last 5 years required for participation in the PROPELS study

#### Participant invitation

Eligible individuals identified as having an HbA1c or blood glucose value within the prediabetes category (see Table 1) within the last 5 years are sent an invitation letter, a brochure about the study, and a reply slip. For those recruited directly from primary care, the invitation letters are sent by the primary care practice where the search was conducted. For those recruited from existing databases, the invitation is sent from the Principal Investigator of that study. Individuals who are interested in taking part are asked to return a reply slip directly to the PROPELS research team. Interested participants are sent the full study patient information sheet along with a confirmation letter and an appointment is arranged for a baseline visit.

### Eligibility criteria

Patients are eligible for the trial if they are:

- Aged 40–74 years old for white European, or aged 25–74 years old for South Asian
- Have a previously recorded plasma glucose or HbA1c value in the prediabetes range within the last five years (see Table 1)
- Have access to a mobile phone, and willing to use it as part of the study

Participants are excluded from the trial if they are:

- Unable to take part in ambulatory-based activity
- Pregnant
- Involved in other related intervention studies
- Diagnosed with diabetes, or diabetes detected at baseline visit
- Unable to understand basic written and verbal English
- Unable to give informed consent

### Algorithm of exclusion for participants found to have T2DM at baseline

At Leicester anyone who has an HbA1c in the diabetes range (see Table 1) at baseline is recalled for a second confirmatory test, and if diabetes is confirmed they are referred back to their physician for routine care. At Cambridge the participant’s primary care physician is informed of the need to confirm diagnosis as appropriate. Individuals found to meet the WHO (2011) [40] criteria for diagnosis of diabetes are excluded from the study.

### Algorithm for inclusion for participants found to have normal glycaemia at baseline

All individuals found to have normal glycaemia at baseline are included in the study provided they meet the inclusion criteria requiring a historical blood glucose level within the prediabetes range within the previous 5 years.

### Randomisation

Once baseline data have been collected, participants are randomised (stratified by sex and ethnicity) using an online randomisation tool hosted at the University of Leicester Clinical Trials Unit. Individuals are randomised (1:1:1) to the control group, the Walking Away Group or the Walking Away Plus Group. The exception to this is individuals recruited from the same household, who are randomised to the same group. Participants are informed of their allocated treatment after baseline measures are completed. Study allocation is concealed from the study measurement teams conducting the 12-month and 48-month follow-up.

## Study interventions

### Detailed advice leaflet (Control Group)

Participants in the control group receive an advice leaflet detailing the likely causes, consequences, symptoms and timeline associated with prediabetes, as well as information about how physical activity can reduce the risk of developing T2DM. This leaflet is informed by Leventhal’s Common Sense Model [41], which also underpins the structured education programme. Participants also continue to receive standard care from their GP.

### Structured education programme followed by annual group maintenance sessions (Walking Away Group)

Participants are given the same advice leaflet as the control group and invited to attend an updated version of the PREPARE structured education programme within three months of their baseline clinic visit, titled Walking Away from Type 2 Diabetes. Participants are also offered annual maintenance education sessions, revisiting the key messages of the initial Walking Away programme, and discussing any benefits and barriers they have experienced to increasing their physical activity.

The Walking Away programme fully incorporates the curriculum and content of the successful PREPARE programme [32, 37], but was renamed and updated to incorporate current terminology and guidance [6] and now includes an accredited educator training and quality assurance pathway. The PREPARE programme has previously been shown in a single centre RCT to increase ambulatory activity by 2000 steps per day compared to control conditions over a 12-month period when combined with pedometer use with sustained improvements in glucose levels. [32, 37]

The programme is delivered by two trained educators to groups of up 10 participants (who are welcome to bring a guest). The content of the programme, examples of activities and the underlying theoretical structures are presented in Table 2. The key aim of the programme is to increase participants’ physical activity predominantly through increased walking, and to prompt the use of self-monitoring and feedback using pedometers. During the programme participants use their daily habitual step count (measured at baseline prior to the education programme) to set personalised activity goals. They are provided with a pedometer (Yamax SW200) as a self-monitoring tool. Typically, sedentary participants are encouraged to increase their activity levels by at least 3000 steps per day, equivalent to around 30 minutes of walking. Those achieving more than 6000 steps per day are encouraged to try to reach at least 9000 steps per day, an amount that is likely to include 30 minutes of walking activity in addition to usual daily activity [32]. Those achieving more than 9000 steps per day are encouraged to at least maintain their current activity levels and develop further goals should they wish to. Goal attainment is encouraged through the use of proximal objectives, such as increasing ambulatory activity by 500 steps per day every two weeks. Participants are encouraged to make an action plan detailing where, when and how their first proximal goal will be reached, to repeat action planning for each new proximal goal, to wear their pedometer on a daily basis and to self-monitor their ambulatory activity using a specifically designed steps-per-day diary. A full description of the rationale, development and efficacy of the work underpinning Walking Away are detailed elsewhere [32, 42].

**Table 2:** Outline of the Walking Away programme (delivered to the Walking Away group and the Walking Away Plus group)

Module:	Main aims:	Example activity:	Theoretical underpinning:	Time weighting
Patient Story	Give participants a chance to share their knowledge and perceptions of prediabetes and highlight any concerns they may want the programme to address.	Participants are asked to share their story, how they were diagnosed with prediabetes and their current knowledge of prediabetes	Common Sense Model [67]	15 % (30 minutes)
Professional story and risk communication	Use simple non-technical language, analogies, visual aids and open questions to provide participants with an overview of healthy glucose metabolism, the aetiology of prediabetes and diabetes, and the risk factors and complications associated with elevated blood glucose levels (cholesterol, blood pressure, cardiovascular risk etc.). Support participants to consider how their individual risk factors (modifiable and non-modifiable) can stack up, identify their own personal risk factors, and identify options to reduce their risk	1) The following model for insulin resistance is used: Glucose moves from the blood into cells to be used as energy via a door with a lock on it. Insulin keys are used to open the lock; insulin resistance occurs when the cell locks get rusty.	Common Sense Model [41], Social Cognitive Theory [68], Dual Process Theory [69]	35 % (60 minutes)
		2) Using resources participants are encouraged to share their knowledge of their risk factors for developing T2DM, plot their own personal risk factors and work out which risk factors they can personally alter.		
Diet	Give participants an accurate understanding of the link between dietary macro-nutrients and metabolic dysfunction	Participants are asked to group food models into their dominant macro-nutrient groups (i.e. carbohydrate, fat, protein). Fats and oils are divided into saturated, polyunsaturated and monounsaturated categories.	Social Cognitive Theory [68] Dual Process Theory [69]	10 % (20 minutes)
Physical activity	Use simple non-technical language, analogies, visual aids and open questions to help participants: identify how physical activity improves glucose control; understand the current physical activity recommendations and how these relate to steps per day; explore options for incorporating physical activity (primarily walking) into everyday life; identify barriers to exercise; form action plans; encourage participants to use their provided physical activity diaries and pedometers; and set personal step per day goals.	1) Participants are encouraged to share their knowledge of the various exercise recommendations and to work out how each recommendation may affect their health.	Social Cognitive Theory [68]	40 % (70 minutes)
			Implementation Intentions [70] Dual Process Theory [69]	
		2) Participants are provided with a physical activity diary and pedometer and helped to set their first action plan.		

Following the initial group session, participants are offered annual group-based maintenance sessions at 12, 24 and 36 months, each lasting 2.5 hours. These maintenance sessions are designed to re-visit the key messages of the first session, and build self- efficacy through the sharing of successes, problem solving barriers, goal setting and self-monitoring using pedometers. This mode of delivering behaviour change maintenance is designed to be directly relevant to primary care pathways where annual clinical follow-up of those with a high risk of chronic disease, such as prediabetes, is recommended [5, 6].

### Structured education programme plus follow on support (Walking Away Plus)

Participants receive the same advice leaflet, Walking Away structured group education programme and annual maintenance sessions. In addition they are introduced and given access to a highly tailored text-messaging follow-on service designed to support behaviour change and pedometer use with additional telephone calls from educators that are initiated after attendance at the Walking Away programme. Details of the text messaging and telephone follow-on support service are detailed in Table 3 and have been submitted for publication elsewhere. In brief, approximately one week after the Walking Away session, an educator telephones the participant to help them identify a short- and long-term step goal and action plan for the next six months, and to elicit information on tailoring variables including confidence in increasing physical activity, previous experience of physical activity and potential mobility issues that prevent walking being the primary activity. The educator records the information in an online form and saves it on a database for use by the text-messaging programme. A key feature of the text-messaging and pedometer support is that participants are prompted to text in their weekly step count. They then receive automated feedback by text message tailored to the extent to which they achieved their goals, in relation to the tailoring variables. Participants also receive text-messages using evidence-based behaviour change techniques and a six month telephone call to review their goals and action plans, prompt problem solving and provide social support. The key techniques used in the follow-on support are goal setting (behaviour), action planning, self-monitoring, goal review, problem-solving and social support [43]. The intensity of the ‘follow-on support’ varies over the course of the year (see Table 3) and is repeated over the four years of the study. Participants who do not wish to receive text-messages can opt out at any time.

**Table 3:** ‘Follow on’ support for the Walking Away Plus Group, repeated over the four years of intervention

Time point from education attendance	Type of contact and frequency	Content (behaviour change techniques and their delivery)
0 months	First group session (3 hrs)	• As the ‘Walking Away’ group plus extra 15–20 minutes at end of the session to explain the follow-on support and what to expect over the next 12 months in terms of text-messaging, pedometer support and telephone calls.
		• One week of self-monitoring (using the pedometer and activity diary) and text messages prompting participant to ‘text in’ their weekly step total at the end of the week (‘baseline’ steps).
1 week	First telephone call from educator (15 minutes)	• Educator prompts participant to set an action plan and personal short term and long term goals informed by the baseline steps, and asks participant about their confidence to achieve goals and previous levels of physical activity. Educator records this information on an online form which saves to a database for use in tailoring subsequent text-messages.
0-2 months	Text message contact (1–3 per week)	• Participant monitors activity (pedometer step counts) each week, using a pedometer, an activity diary and a converter to translate activities other than walking into steps.
		• Participant receives text-messages asking them to ‘text in’ weekly step count total.
		• Participant receives feedback by text-message tailored to goal achievement, confidence, and previous physical activity levels.
		• Participants who do not make progress with goals receive ‘problem solving’ texts, asking them to text in barriers, followed by tailored replies.
2-6 months	Text message contact (one per week)	• Weekly tailored messages targeting attitudes and beliefs, motivation, self-efficacy and self-regulation of PA behaviours.
		• Participant is asked to self-monitor and record steps for 1 week and text in weekly amount (ahead of 6 month telephone call)
6 months	Telephone contact; 15 minutes	• Educator gives feedback on goal progress, and reviews goals.
		• Educator prompts problem solving in relation to barriers.
		• Educator identifies and highlights benefits experienced.
		• Educator discusses whether experiences of behaviour change are satisfying and reinforcing.
		• Educator provides social support.
		• Educator prompts continued goal setting and action planning.
7-12 months	Text message contact once per month	• Monthly tailored messages target attitudes and beliefs, motivation, self-efficacy and self-regulation of physical activity behaviours.
		• Participant is asked to self-monitor and record steps for 1 week and text in weekly amount (ahead of 12 month group education session)
OPTIONAL	Telephone contact; 15 minutes	• Educators call participants who do not respond to text requests for step counts, to encourage participation and solve any problems.
12 months	Walking Away maintenance session; 2.5 hours	• See Walking Away group (Table 2).

This annual structure is repeated each year following each group education maintenance session for the 4 year intervention period

### Educator recruitment, training and quality assurance (intervention fidelity)

Educators can be registered health care professionals (e.g. nurse, dietician) or a suitable non-registered professional (e.g. Health Trainer). Across the study sites, 16 educators have been recruited from a range of registered and non-registered Health Care Professionals from local healthcare providers and other appropriate settings. This mix of personnel was included in order to make the study as pragmatic and generalisable as possible to routine health care systems. To ensure that all interventions are delivered by educators as planned, a fidelity standard operating procedure has been put in place. All educators attended an initial two day training course to ensure that they understood the theories and philosophy that underpin the Walking Away programme and the content and resources used within it. All educators were given a written curriculum to support their delivery of the programme and given the opportunity to practise delivery of the programme. Quality assurance of delivery is undertaken to assess educational style and content using established tools used within routine care through the DESMOND Collaborative [44]. Educators receive structured and instructive feedback from their assessor and key goals and action plans are developed in order to help them improve their performance. Prior to the delivery of the one year intervention, educators received training to be able to deliver the Walking Away Maintenance session which is also supported by a written curriculum.

Educators attended additional training in delivering the telephone calls to participants in the ‘Walking Away Plus’ group. The training was supported by an extensive curriculum outlining the contents (behaviour change techniques, patient-centred communication skills) and mode of delivery of the follow-on support. The curriculum also contains standardised scripts that educators are asked to follow, an explanation of the intervention fidelity procedures, and standardised reflection sheets and checklists to promote and assess fidelity of the phone calls. Educators are asked to audio-record the telephone calls, listen back to a sample, complete the checklists, and discuss these with the intervention lead. The number of calls assessed and their frequency depends on the competence level of the individual educators as well as the year of intervention delivery. Monitoring the delivery of the text-messaging system is done on a weekly basis by examining automatically generated lists of messages sent and received.

### Study outcomes

Data collection clinics are run by research nurses in the Leicester Diabetes Centre, the MRC Epidemiology Unit, Cambridge and other local community centres and clinic areas. All staff have been trained in study procedures and data are collected following standardised operating procedures. Written informed consent is obtained from all participants prior to the commencement of data collection. Details of all clinical assessments and outcome measures are provided in Table 4. Participants are sent a letter with details of selected clinical results after each visit, and their results are also sent to their general practitioner.

**Table 4:** Participant assessments at each time point

Clinical Assessment	0 months	12 month	48 months
Family history of disease	X	X	X
Medication status	X	X	X
Smoking status	X	X	X
Muscular/skeletal injury	X	X	X
Blood pressure*	X	X	X
Height*	X		X
Weight*	X	X	X
Waist circumference*	X	X	X
Arm and leg length	X		
Body fat percentage	X	X	X
Fasting and 2-hr glucose and insulin (Leicester only)	X	X	X
HbA_1c_*	X	X	X
Lipids*	X	X	X
Urea & Electrolytes*	X	X	X
Liver Function Tests*	X	X	X
7 Day Step Count & Physical Activity (accelerometer)	X	X	X
Recent Physical Activity Questionnaire (RPAQ)	X	X	X
Dietary questions	X	X	X
Brief Illness Perceptions Questionnaire (BIPQ)		X	X
Physical activity self-efficacy	X	X	X
Enactment of techniques (Groups 2 & 3 only)		X	X
EQ-5D; SF-8	X	X	X
Hospital Anxiety and Depression Scale (HADS)	X	X	X
Sleep	X	X	X
Neighbourhood Environment Walkability Survey (NEWS)	X		
Use of health resources	X	X	X

*Results of these assessments are sent to the participant and their primary care physician

### Primary outcome

The primary outcome measure is change in ambulatory activity (steps per day) at 48 months, assessed by accelerometer (Actigraph GT3X+) with an intermediary assessment at 12 months. Participants are asked to wear the accelerometer on a waistband (in the right anterior axillary line) for seven consecutive days during waking hours following their baseline and follow-up visits. In addition, participants are asked to fill in a log sheet each day that they wear the accelerometer indicating time the accelerometer was taken off at night, time they went to sleep, time they woke up and time the accelerometer was attached. At the end the seven days of wear, participants are asked to return the accelerometer and the log sheet to the research team in a pre-paid envelope. Raw acceleration data are captured and stored at 100 Hz. Data processing will be undertaken on commercially available analysis tools. At least three days valid wear is required to count as a valid recording. Non-wear time is determined by one hour or more of consecutive zero counts. Due to the potential for bias between groups in factors used to acquire valid accelerometer data, average wear time and the number of valid days will be included as covariates in the analysis.

### Secondary outcomes

#### Accelerometer

The accelerometer used to measure the primary outcome, detailed above, is also used to assess the number of censored steps taken per day, defined as steps taken above an intensity used to distinguish between purposeful and incidental ambulation [45]. In addition, commonly used cut-points will be used to distinguish between time spent sedentary and in time spent in light-, moderate, and vigorous-intensity physical activity [46]. We will also use this data to assess compliance to the physical activity recommendation of undertaking at least 150 minutes of moderate-to-vigorous intensity physical activity per week in bouts of at least 10 minutes.

#### Time spent lying, standing, sitting and postural transitions

Participants are also asked to wear an activPAL3™ device for the same seven days as the ActiGraph accelerometer. This is a small slim thigh worn monitor that uses accelerometer-derived information about thigh position to determine body posture (i.e., sitting/lying, standing and stepping). The activPAL3™ is initialised using manufacturer’s software with the default settings (i.e., 20Hz, 10s minimum sitting-upright period) and participants are asked to wear the device continuously (24 hours/day). The device is covered with a nitrile sleeve and fully wrapped in one piece of waterproof dressing (Hypafix Transparent) to allow participants to wear the device during bathing activities. The activPAL3™ is worn on the midline anterior aspect of the upper thigh and secured using hypoallergenic waterproof dressing (Hypafix Transparent).

#### Recent Physical Activity Questionnaire (RPAQ)

Self-reported physical activity is measured using the RPAQ. This assesses physical activity across four domains (domestic, recreational, work, commuting) over the previous month. The domestic section contains questions regarding computer use, TV-viewing and stair climbing at home. The questions in the recreational domain ask about frequently performed activities, including frequency and duration. The work domain examines the level of physical activity associated with their current employment, and commuting assesses four modes of usual transport: walking, cycling, car, and public transport. It has shown moderate-to-high reliability for physical activity energy expenditure, and good validity for ranking individuals according to their time spent in vigorous intensity physical activity and overall physical activity energy expenditure [47]. Participants also complete the Neighbourhood Environment Walkability Survey (NEWS) which captures the environmental context in which participants live and will be used to assess environmental determinants of physical activity and physical activity behaviour change [48, 49].

#### Biochemical variables

Standard biomedical outcomes are assessed by venous sampling. These consist of HbA1c, lipid profile (triglycerides, HDL, LDL, total cholesterol), urea and electrolytes (sodium, potassium, urea, creatinine) and liver function tests (albumin, total bilirubin, alkaline phosphatase, alanine transaminase).

At the Leicester site only, participants are assessed for fasting and 2-hour post challenge glucose and insulin levels using an Oral Glucose Tolerance Test (OGTT). The OGTT results will be used to provide greater clinical insight into how the promotion of physical activity affects metabolic health in prediabetes, given 2-hour glucose and insulin levels better reflect peripheral insulin sensitivity. Given that the new WHO guidelines [40] have led to local clinical practice basing diagnosis of T2DM on HbA_1c_ criteria, the routine use of the OGTT has been phased out of primary care. Therefore, in order to comply with local guidelines and to avoid confusing clinical management strategies in recruited practices, OGTT samples taken in Leicester are frozen and will be analysed after the final study visit (48 month) and not form part of a diagnosis of diabetes. The OGTT involves a fasting blood sample being taken from the patient before they are then given a glucose load of 75 g. A second sample is taken 2 hours later. Fasting and 2-hour plasma glucose and insulin samples are taken at each clinical visit and stored in a −80 °C freezer using standardised, stable methodology within the Leicester Diabetes Research Centre.

#### Genetics

A blood sample for genetic analysis is also collected in those who provide their consent. The aim of this sample will be to investigate group level associations and interactions of physical activity, obesity and genes in the development of T2DM. The genetic assessments will be focused on genes for which there are biological plausibility for interaction.

#### Anthropometric and demographic variables

Body weight, body fat percentage, height and waist circumference are measured to the nearest 0.1 kg, 0.5 %, 0.5 cm and 0.1 cm respectively. Waist circumference is measured using a soft tape mid-way between the lowest rib and iliac crest. Arterial blood pressure is obtained from the right arm of the seated participant. Three measurements are taken and an average of the last two measurements will be used. Total upper arm length, forearm length, total leg length and lower leg length are measured on the left side of the body. Information on ethnicity, medication history, current smoking status, family history of diabetes in first and second degree relatives, and muscular/skeletal injury that prevents physical activity are obtained by self-report.

#### Cardiovascular risk

Cardiovascular risk is calculated using the Framingham risk calculator.

#### Self-reported dietary behaviour

In order to capture dietary behaviour, two short questionnaires used in previous research studies by our group are administered to the participants for self-completion. These questions are based on dietary questionnaires developed for the EPIC (European Prospective Investigation of Cancer and Nutrition) study and the international NAVIGATOR (Nateglinide And Valsartan in Impaired Glucose Tolerance Outcomes Research) study [50, 51].

#### Sleep

Participants self-report on two questions concerning sleep duration over the past 24 hours and over a usual week. There is accumulating evidence for an association between short sleep duration (<6 hours per 24 hours) and long sleep duration (≥10 hours per 24 hours) and metabolic dysfunction [52].

#### Health related quality of life

Health related quality of life data is measured using the European Quality of Life-5 Dimensions (EQ-5D) [53] and the Short Form (SF-8) Health Survey [54]. The EQ-5D is a standardized questionnaire that was developed for use as a measure of health outcomes and defines health in terms of five dimensions: mobility, self-care, usual activities, pain or discomfort, and anxiety or depression. It is widely used to calculate ‘quality adjusted life years’ (QALYs) which are essential to cost-effectiveness analysis. The SF-8 is a self-administered questionnaire measuring eight health domains (general health, physical functioning, role physical, bodily pain, vitality, social functioning, mental health and emotional roles) with eight questions. The standard (4 week) recall format will be used. Data from SF-8 is represented as a physical component score and a mental component score.

#### Depression and anxiety

Depression and anxiety are measured using the Hospital Anxiety and Depression (HADS) Scale, to produce independent subscales for anxiety and depression [55].

#### Health resources

A health resources questionnaire records the number of times over the past 12 months that the participant has seen a health care practitioner such as a GP, nurse or other health workers, and the number of times they have been to hospital. In addition, the contact and costs associated with the intervention will be captured by the research team. These costs will be inputted into the cost effectiveness analysis of the intervention.

### Process measures

#### Perceptions of diabetes risk

Perceptions and perceived knowledge of diabetes risk is measured at 12 and 48 months using the validated Brief Illness Perceptions Questionnaire (BIPQ) [56]. This eight item instrument uses an 11 point Likert scale (0 = no effect, 10 = complete effect) to measure five cognitive diabetes risk representations (consequences, timeline, personal control, treatment control, and identity), two emotional representations (concern and emotion) and risk comprehensibility (perceived knowledge).

#### Self-efficacy in relation to physical activity

Self-efficacy is measured at baseline, 12 and 48 months. Six items measure participants’ confidence in their ability to undertake any form of moderate- to vigorous-intensity physical activity for 10 minute periods, increasing incrementally from 10 minutes to one hour each day. Items use a 100 % confidence rating scale (where 0 % = no confidence, and 100 % represents complete confidence). An overall score is calculated by summing the efficacy scores for each time period and dividing by the number of time periods.

#### Enactment of the behaviour change techniques

Participants’ use of behaviour change techniques included in the Walking Away and Walking Away Plus groups is assessed at 12 and 48 months. A 5-point Likert scale (where 1 = most of the time and 5 = never) assesses how often participants set goals, form action plans, use a pedometer, complete an physical activity log, are aware of their activity levels, and are trying to be more physically active.

#### Uptake and adherence to Walking Away and Walking Away Plus interventions

Measures of uptake and adherence to the intervention groups will include: 1) group session attendance to Walking Away, 2) group session attendance to the annual maintenance sessions at 12, 24 and 36 months 3) proportion of phone calls completed, 4) number of participants who registered for the text messaging service, 5) number of STOP messages received for test messaging (i.e. number opting out of the text messaging and pedometer support), 6) proportion of intended texts sent, and 7) number of step count texts received from participants relative to the number of requests they are sent (engagement).

### Qualitative process evaluation

A process evaluation will be conducted using a combination of ethnographic methods including observations of education sessions and individual interviews. Briefly, we will observe a sample of Walking Away sessions and undertake interviews with a sample of participants from all three trial groups. Analysis will be informed by the constant comparative method; the focus will be exploring whether and how participants engage with each component of the intervention(s).

### Sample size

#### Primary outcome

For 1-beta = 0.8, alpha = 0.025 (allowing for 2 a priori comparisons against control conditions), SD = 4000 steps/day and a drop-out of 30 %, we require 436 per group (1308 in total) to detect a 1000 steps/day difference in change in ambulatory activity (equivalent to 10 mins walking/day or 70 mins walking/week) between the intervention groups and control group. Assuming 25 % of participants in the total cohort are SA we have an 80 % power to detect around a 2000 steps/day difference when comparing two intervention comparisons to the control group (alpha = 0.025) in the SA population.

Several intervention studies with a follow-up of between 3 to 12-months reported a standard deviation of change in ambulatory activity of around 3000 to 4000 steps per day in individuals with T2DM, prediabetes or in sedentary individuals [32, 57–59].Therefore we have anticipated a standard deviation of change of 4000 steps per day. An intervention effect of 1000 steps was considered the minimum clinically significant difference between groups and equates to around a 4 % difference in the risk of cardiovascular morbidity and mortality [13].

#### Secondary outcome

Given that around 95 % of the general population fail to meet the Chief Medical Officer’s physical activity guidelines when measured objectively by accelerometers [60], this study also allows for the 10 % difference in those meeting the current physical activity recommendation to be detected at follow-up based on 1-beta = 0.8, alpha = 0.025. Consistent with the calculation for ambulatory activity, this study has sufficient power (1-beta = 0.8, alpha = 0.025) to measure a 10 minute/day difference in change in the time spent in moderate-to-vigorous intensity physical activity based on previous work undertaken by our group [16].

This study has sufficient (1-beta = 0.8, alpha = 0.025) power to allow for clinically meaningful differences for change in the biochemical measures to be detected in the entire study cohort and after stratification by ethnicity; fasting glucose (0.3 mmol/l), 2-h glucose (1 mmol/l) and HbA_1c_ (0.25 %).

Furthermore assuming a conversion rate to T2DM in the control group of at least 24 % over the course of the entire study (4 years), we will have an 80 % power to detect a 40 % reduction in the relative risk of T2DM in both intervention groups compared to the control group. The estimated conversion rate is at the lower level reported for traditionally defined prediabetes [7, 61]. We anticipate that the inclusion of an HbA_1c_ defined prediabetes in this study will act to marginally lower the conversion rates, whilst the inclusion of a large South Asian group will act to increase the conversion rates.

### Data analysis

Analysis will involve two *a priori* comparisons; both intervention groups will be compared to the control group. Should any of these comparisons reveal a significant difference, then a third *a priori* comparison will be undertaken by comparing the difference between intervention groups - this will be included as a secondary analysis. Multiple linear regression analysis will be used to investigate the differences in the change in physical activity level achieved between groups at 48 months, after adjusting for potential areas of bias between groups, valid accelerometer wear time and the number of valid wear days. Analysis will be conducted on the cohort as a whole and stratified by ethnic group; interaction terms will be used to quantify the effect of ethnic group. We will also use interaction terms to assess whether the effects of the interventions are modified of gender, age, ethnicity, family history of T2DM or whether prediabetes status was confirmed at baseline; significant interactions will followed by stratified analysis. We will also assess change in physical activity at 12 months as a secondary outcome. The primary analysis will be based on analysing those with complete data at each time point in the group to which they were randomised. A sensitivity analysis will be undertaken to assess the impact of imputing missing data through multiple imputation and through a per-protocol analysis by removing those that failed to attend the initial Walking Away session in both intervention groups; per-protocol analysis for the Walking Away Plus group will be defined as removing those that that failed to attend the initial Walking Away session OR those that failed to register for or actively stopped their text messaging support via the STOP function within the first 2 months.

Analysis of secondary biochemical and anthropometric outcomes will be analysed using the same strategy and at the same time-points as that described for the primary outcome. Differentials in the time to T2DM between groups will be plotted using the Kaplan–Meier method for comparing survival curves; the log rank test will be used to assess for differences between the groups.

### Health economics

We will undertake a costing exercise to determine the cost of delivering the initial interventions covering expenditure such as educator time, educator training and quality assurance. In addition we will determine the cost of the follow-up maintenance support group-sessions and the staff and other costs of the individually tailored telephone and text messaging package for maintenance support. Resource use incurred will be costed using actual costs in the trial and/or standard references for unit costs such as Unit Costs of Health and Social Care [62]. In addition to the primary endpoint, we will analyse the within-trial impact of the interventions during the trial on other outcomes that are pertinent to the long-term economic analysis, i.e. use of antihypertensives and lipid-lowering therapies, blood pressure, health utility and incidence of T2DM.

Long-term costs and benefits of the interventions will be evaluated through a combination of the within-trial outcomes and decision-analytic modelling to simulate long-term incidence ofT2DM, microvascular complications arising from T2DM and cardiovascular events. Specifically for progression to T2DM, estimating long-term progression will require a statistical model built partly from incidence data from the trial. The underlying incidence curve will be based on rates of progression in the control arm, and a survival model with time-varying hazards will be built to demonstrate the effect of a unit change in physical activity on risk of T2DM over time. This will allow the impact of alternative assumptions about the degree of maintenance of physical activity beyond the 4-year follow-up period to be modelled. The underlying progression of T2DM beyond the 4-year follow-up will be estimated by the above 4-year survival curve and assumptions about medium-term maintenance of physical activity, but also informed by the trajectory of survival curves from long-term diabetes prevention studies such as the Finnish Diabetes Prevention study [63].

An important input for the modelling will be the effect of increasing physical activity on cardiovascular risk. The relationship between changes in physical activity and cardiovascular risk will be incorporated into an existing decision-analytic model of prevention of diabetes. This relationship will be informed by a recent study that specifically calculated the effect of change in ambulatory activity (steps per day) on cardiovascular disease risk [13]. The model will be chosen from existing models used previously for work for NICE [64] on prevention of diabetes or developed as part of the School for Public Health diabetes prevention theme within ScHARR, funded by the National Institute for Health Research (NIHR). The economic model aggregates the costs of the intervention, prescribed medications, therapy, the costs of one-off treatments (e.g. cost of amputation), and on-going treatment of complications (e.g. treatment following stroke). The cardiovascular risks of participants with prediabetes, or with diabetes will be estimated using the UKPDS risk engines [65, 66]. A further adjustment will be made so that the risk can be adapted for the South Asian population using evidence advised by clinical colleagues.

Separate evaluations will be undertaken for the overall group and for the South Asian subgroup. Cost-effectiveness will be reported in terms of incremental costs and QALYS and the incremental cost-effectiveness ratio. Uncertainty around the results will be explored through probabilistic sensitivity analysis and related techniques for identifying the most important drivers of uncertainty, which will be presented on a cost-effectiveness acceptability curve and a cost-effectiveness plane.

### Trial Steering Committee (TSC) and Data Monitoring and Ethics Committee (DMEC)

The trial is overseen by TSC comprising an independent chair, independent clinical and academic members and the principal investigator. This committee is responsible for the overall management and oversight of the trial. The steering committee are blinded to all information regarding treatment assignments until after the database is locked for final analysis.

A fully independent DMEC reports to the TSC. This comprises an independent chair and a statistician. The DMEC are responsible for the interests of participant safety and data integrity. The DMEC will undertake safety data reviews every nine months, unless otherwise deemed necessary. In addition the DMEC will also review analysis plans.

## Discussion

This trial will provide important insight regarding the long term effectiveness and cost-effectiveness of a group-based structured education programme for physical activity targeting those with prediabetes within primary care, and the effectiveness of a highly-tailored and evidence-based text-messaging and telephone follow-on system designed to support ongoing behaviour change and pedometer use. NICE guidelines on diabetes prevention recommend the use of group-based lifestyle intervention for prediabetes as the cornerstone of any diabetes prevention pathway [6], so it is important to investigate the use of structured education within the prevention domain. It is imperative that any intervention programme is carefully evaluated for both clinical and cost-effectiveness, as well as its capacity to be easily implemented into primary care. Whilst both the effectiveness and cost-effectiveness of structured education for those diagnosed with T2DM have already been established [34, 35], and is recommended by NICE as best clinical practice for the management of T2DM [33], there is a lack of translational research within the context of diabetes prevention. Consequently this limits the capacity of commissioners and policy makers to make informed evidence-based decisions regarding the implementation of national diabetes prevention programmes within primary care. Furthermore there is a need to establish what the optimal levels of ongoing support are to ensure any behaviour change is maintained, taking into account the limited resources available in most healthcare settings. This study evaluates highly-tailored, evidence-based text-messaging tool which requires few resources and time, and thus will help address both of these limitations.

The Walking Away from Diabetes structured educational programme and the follow-on text-messaging and telephone support system have been specifically designed to be translated into primary care. This type of lifestyle intervention in prediabetes could be easily added to other programmes currently on offer for the management of type 2 diabetes, thereby allowing a range of structured education packages and evidence-based mHealth interventions to be offered, to best serve the needs of the diabetes pathways within primary care.

## Trial status

Recruitment started in January 2014 and is still ongoing.

## Abbreviations

BIPQ: Brief illness perceptions questionnaire
DESMOND: Diabetes Education and Self-Management for Ongoing and Newly Diagnosed
DMEC: Data Monitoring and Ethics Committee
EPIC: European Prospective Investigation of Cancer and Nutrition
EQ-5D: European Quality of Life-5 Dimensions
HbA1c: Glycated haemoglobin
prediabetes: Impaired glucose regulation
NAVIGATOR: NateglinideAnd Valsartan in Impaired Glucose Tolerance Outcomes Research
NICE: National Institute of Health and Care Excellence
OGTT: Oral glucose tolerance test
PA: Physical activity
QALY: Quality adjusted life years
PREPARE: Prediabetes Risk Education and Physical Activity Recommendation and Education
PROPELS: The PRomotionOf Physical activity through structured Education with differing Levels of ongoing Support for those with prediabetes
SF-8: Short form 8
T2DM: Type 2 Diabetes Mellitus
TSC: Trial steering committee
WHO: World Health Organization
